# *Methanosarcina acetivorans* requires methanol:coenzyme M methyltransferases for ethane formation from ethanol

**DOI:** 10.1007/s10482-025-02165-x

**Published:** 2025-09-20

**Authors:** Tejas Somvanshi, Mai Anh Tran, Jichen Bao, Silvan Scheller

**Affiliations:** Department of Bioproducts and Biosystems, School of Chemical Engineering, Aalto University, 02150 Espoo, Finland

**Keywords:** Ethane, Substrate promiscuity, Ethyl-transfer, Methyltransferases, *Methanosarcina*, Corrinoids

## Abstract

Corrinoid-dependent enzymes either catalyze methyltransfer reactions, or they generate substrate radicals using adenosylcobalamin for subsequent rearrangement reactions. The corrinoid-dependent methyltransferases are present in all domains of life and assumed to be exclusive for methyl-groups. In *Methanosarcina*, however, trace ethane production from ethanol has been shown in vivo*,* which led to the hypothesis that corrinoid-dependent methanol-specific methyltransferases are promiscuous towards also accepting ethyl-groups. Here, we show that the conversion of ethanol to trace amounts of ethane in *Methanosarcina acetivorans* involves homologous reactions of the known methanol-to-methane metabolism. The methanol methyltransferase (MtaB) activates ethanol and loads the ethyl-group onto the corrinoid-containing methyl-accepting protein (MtaC). Besides MtaCB, substrate promiscuity in corrinoid:coenzyme M methyltransferase (MtaA) and methyl-coenzyme M reductase (Mcr) are required to grant the microbe the capacity for ethane production. We show that the MtaCB subunits of *M. acetivorans* can activate ethanol, however, the ethane yields compared to methane are ca. 3 orders of magnitude lower. The ethyl-transfer capability was confirmed for each of the three MtaCB isozyme by quantifying the amount of ethane produced by *mtaCB* double deletion strains during growth in ethanol-supplemented media and in resting-cell suspensions. Ethane formation requires the cells to be grown on methanol to trigger the expression of the *mtaCB* genes, and detectable ethane formation starts only after all methanol has been consumed. Demonstrating that corrinoid-dependent methanol-specific methyltransferases process ethyl groups extends the pool of reactions to be considered in metabolic networks and suggests possible routes for biogenic ethane in nature.

**Supplementary Information:**

The online version contains supplementary material available at 10.1007/s10482-025-02165-x.

## Introduction

Corrinoid-dependent enzymes are broadly classified into two types- methyltransferases that do not use *S*-adenosylmethionine (which contains an activated methyl group) but rather use a methyl group loaded on a cobalt atom of the corrinoid group, and enzymes that catalyze generation of substrate radicals and subsequent rearrangement reactions using adenosylcobalamin (Matthews 2001, 2009). Corrinoid-dependent methyltransferases are present in all three domains of life and are part of the energy metabolism of many anaerobic organisms (Matthews et al. 2008). The cobalt center of the cobalamin acts as a carrier for the methyl groups, cycling between the Co(I) and methyl-Co(III) state. Occasionally, the Co(I) form is oxidized to an inactive Co(II) state, which requires reductive activation either dependent on *S*-adenosylmethionine or ATP to return to the catalytically active Co(I) state (Matthews et al. 2008).

In all methanogens and in anaerobic methanotrophic archaea, corrinoid-dependent methyltransferases catalyze the transfer of methyl groups in at least one of the following catabolic reactions: (i) between tetrahydromethanopterin and coenzyme M (CoM) in most methanogenesis pathways and in anaerobic methanotrophy (ii) from acetate to tetrahydromethanopterin in aceticlastic methanogenesis (iii) from methylated substrates to CoM in methylotrophic methanogenesis (iv) from methoxylated aromatics to tetrahydromethanopterin and (v) from a methylated corrinoids to homocysteine for methionine synthesis (Matthews et al. 2008; Kurth et al. 2021).

Although assumed to be exclusive for methyl groups, corrinoid-dependent methyltransferases have been hypothesized to be promiscuous to also transfer ethyl groups (Loerch and Mallette 1963; Belay and Daniels 1988). Referred to as substrate promiscuity, the enzymes catalyze the same type of reaction, but accept a homologous substrate- in this case ethylated compounds compared to methylated compounds (Hult and Berglund 2007). Notable instances have been in ethionine production and secretion by bacteria (Loerch and Mallette 1963; Röth et al. 2019) and possibly in biogenic ethane formation (Belay and Daniels 1988). While most ethane is formed by thermogenic or abiotic processes, evidence for biogenic ethane has been reported from anoxic slurries and from isotopic signatures typical for biological processes (Oremland 1981; Oremland et al. 1988; Hinrichs et al. 2006; Xie et al. 2013; Musat et al. 2025). The presence of biogenic ethane indicates that there are additional processes to be considered in geomicrobiology (Hinrichs et al. 2006; Musat et al. 2025). Methanogens were putatively the source of biogenic ethane, because anoxic slurries did not produce ethane in presence of methanogenesis inhibitor and pure cultures of *Methanosarcina barkeri* strains showed trace ethane formation in an ethanol-supplemented medium (Oremland 1981; Belay and Daniels 1988). Formation of ethane from ethyl-CoM catalyzed by methyl-coenzyme M reductase (Mcr) via substrate promiscuity has been shown (Goenrich et al. 2004; Scheller et al. 2013). Further evidence that Mcr can convert multi-carbon substrates comes from homologs of Mcr that have been identified in other archaea that catalyze the reversible oxidation of non-methane alkanes (Laso-Pérez et al. 2016, 2019; Chen et al. 2019; Hahn et al. 2020; Zehnle et al. 2023). In these alkane-oxidizing archaea, the “C1 enzyme” Mcr appears to have evolved for catalyzing multi-carbon substrates as its new primary function.

**Fig. 1:**
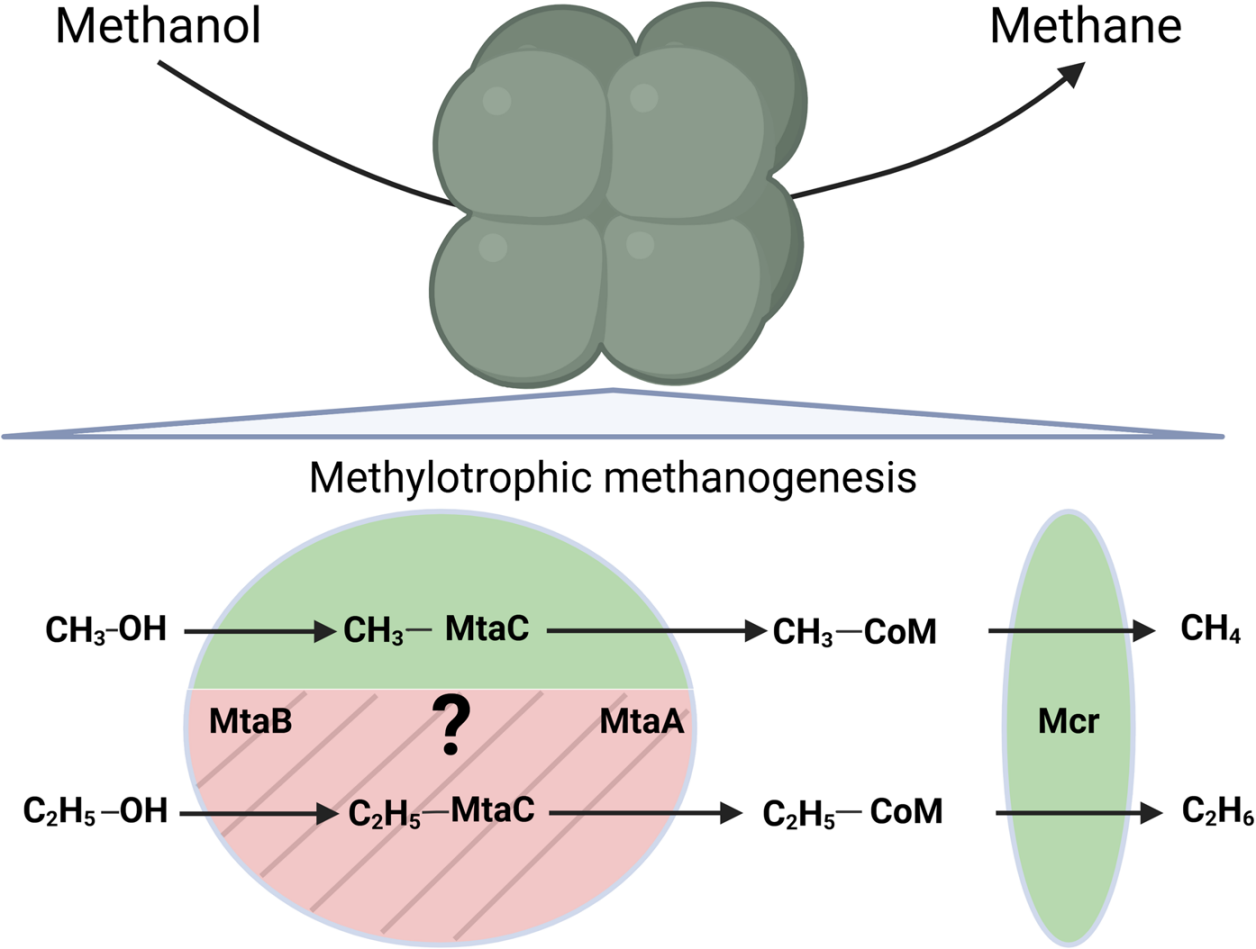
Hypothesized pathway for the conversion of ethanol to ethane. Ethane production follows the homologous pathway as methane production from methanol utilizing the enzymes Mta and Mcr, but the reactions proceed with an ethyl group instead of a methyl group. Methanol methyltransferase (MtaB) activates methanol/ethanol and methylates/ethylates the corrinoid protein MtaC. Corrinoid:coenzyme M methyltransferase (MtaA) transfers the methyl/ethyl group to CoM. Methyl-CoM/ethyl-CoM is then reduced to methane/ethane by methyl-CoM reductase (Mcr) using coenzyme B as the reductant (not shown in figure for simplicity). Green (plain) background marks substrate promiscuity that has been known and published whereas red (striped) marks the hypothesis addressed in this publication. Created in https://BioRender.com

The metabolic pathway responsible for the ethanol-to-ethane conversion has been hypothesized to involve the methanol:coenzyme M methyltransferase (Mta) enzyme present in the methylotrophic methanogens (Fig. 1), because significant ethane production was only seen in methanogens capable of utilizing methanol and when grown using methanol and supplemented with ethanol in the media (Belay and Daniels 1988).

To test the hypothesized pathway (Fig. 1), we focused on Mta from *Methanosarcina acetivorans*, since this organism is a versatile, genetically tractable model methanogen capable of producing methane from methylated substrates such as methanol and methylamines (Sowers et al. 1984; Metcalf et al. 1997; Nayak and Metcalf 2017; Zhu et al. 2023; Bao et al. 2025). *M. acetivorans* encodes 3 isozymes of MtaCB (*mtaCB1- ma_0455-0456, mtaCB2- ma_4391-4392, mtaCB3- ma_1616-1617*), each with varying expression patterns but capable of allowing growth of *M. acetivorans* on methanol (Pritchett and Metcalf 2005; Bose et al. 2006). We tested the impact of ethanol and triethylamine on growth and on ethane production in *M. acetivorans*. Given that promiscuous reactions are often orders of magnitude slower than their primary reactions, ethane was used as the reporter, taking advantage of the high sensitivity of gas chromatography with flame-ionization detector (GC-FID) to quantify trace amounts of ethane. To understand the capability of each MtaCB isozyme for ethyl transfer, deletion strains expressing only one of the three isozymes were grown in presence of ethanol. Resting cell suspensions with protein synthesis inhibitor, similar to previously reported studies (Welander and Metcalf 2005; Schöne et al. 2023), were tested for ethane production to confirm involvement of the proteome that was present during the methylotrophic growth especially the MtaCBs. Finally, ethane production was tested from whole-cell lysate of WT using ethanol, ethylcobalamin and ethyl-CoM to elucidate the possible intermediates in ethane production.

## Materials and methods

### Microbiological and molecular methods

*M. acetivorans* strains (Table 1) were cultured in a high-salt (HS) medium (Sowers et al. 1993) tailored to the specific requirements of the experiment. Briefly, the strains were constructed using a two-step markerless exchange method (Pritchett and Metcalf 2005). The first step consisted of a liposome-mediated transformation of *M. acetivorans* with a non-replicating plasmid that carried the desired *∆mtaCB* allele along with the *hpt* (hypoxanthine phosphoribosyl transferase) and the *pac* (puromycin transacetylase) genes. The *pac* gene gives *M. acetivorans* puromycin resistance, whereas the *hpt* gene makes it sensitive to the purine analogue- 8-aza-2,6-diaminopurine (8-ADP). Puromycin-resistant colonies were then selected. In the second step, 8-ADP was used as a counter selection marker to obtain the colonies for desired strains. The optical density of the cultures was tracked using a Thermo Spectrophotometer at 600 nm. 5 ml cultures were grown at 37 °C in Balch tubes with the gas phase consisting of 50% N_2_/ 20% CO_2_/ 30% of 1% H_2_S in N_2_ at atmospheric pressure (hereafter referred to as gas mix A). The substrates (Sigma) added for in vivo tests were: 60 mM methanol (MeOH) or 20 mM trimethylamine (TMA) with 60 mM ethanol (EtOH) or 20 mM triethylamine (TEA) supplemented where necessary (Table 2). Doubling times were calculated using QURVE (Wirth et al. 2023).

**Table 1:** List of strains used in the study and their corresponding genotypes

Referred in the study as	Genotype	Original designation	Source
WT	*∆hpt*	WWM1	Pritchett and Metcalf (2005)
*mtaCB1*	*∆hpt ∆mtaCB2 ∆mtaCB3*	WWM5	
*mtaCB2*	*∆hpt ∆mtaCB1 ∆mtaCB3*	WWM7	
*mtaCB3*	*∆hpt ∆mtaCB1 ∆mtaCB2*	WWM9	
*∆3mtaCB*	*∆hpt ∆mtaCB1 ∆mtaCB2 ∆mtaCB3*	WWM13

**Table 2:** List of media conditions

Media	Carbon sources	Concentration
MeOH	Methanol	60 mM
TMA	Trimethylamine	20 mM
MeOH + EtOH	Methanol Ethanol	60 mM 60 mM
MeOH + TEA	Methanol Triethylamine	60 mM 20 mM
TMA + EtOH	Trimethylamine Ethanol	20 mM 60 mM
TMA + TEA	Trimethylamine Triethylamine	20 mM 20 mM

### Resting cell suspension assays

30 ml of mid-exponential phase cells grown in 60 mM MeOH or 20 mM TMA with the gas phase consisting of gas mix A were pelleted at 4000 g for 15 min, washed twice with HS media and then resuspended in 6 ml of HS media with 96 mM ethanol and 3 µg/ml puromycin (Invivogen) to block protein synthesis. The mix was then dispensed into 3 Balch tubes (2 ml each), and the gas phase consisted of gas mix A. The tubes were then incubated at 37 °C.

### Biochemical assays

200 ml of mid-exponential phase cells grown in 60 mM MeOH or 20 mM TMA with the gas phase consisting of gas mix A were centrifuged at 3214 g for 15 min, washed twice with HS media and resuspended in 3.0 ml of 50 mM MOPS/KOH (pH 7) buffer. The cells were then lysed by sonication (10 s, 20% amplitude with MS72 probe on ice). The total protein concentration was determined photometrically with the Micro BCA™ Protein assay kit (Thermo Scientific) using BSA standard curve from 0–40 µg/ml. Ethyl-cobalamin, ethyl-CoM and CoB-S–S-CoB were synthesized using reported protocols (Wedemeyer-Exl et al 2008; Scheller et al. 2010; Chadwick et al. 2023). A master mix was prepared for the assay such that the final 1 ml assay consisted of 50 mM MOPS/KOH, 12.5 mM ATP, 0.3 mM hydroxocobalamin, 0.5 mM CoB-S–S-CoB, 90 mM Ti(III)-citrate, 4 mM CoM-SH and 20 mM MgCl_2_ added in the order mentioned. 700 µl of master mix, 200 µl of whole cell lysate and substrates (100 mM ethanol, 0.1 mM ethylcobalamin or 5 mM ethyl-CoM) were added to 13.5 ml glass vials while being kept on ice. The assay volume was 1 ml for all experiments. The headspace of the vials consisted of 100% H_2_ and the reaction was started by incubating the vials at 37 °C.

### GC-FID quantification of methane and ethane

The first gas samples were taken before incubation after setting up either the growth cultures, the cell suspensions, or biochemical assays. The glass vials and the Balch tubes were then incubated at 37 °C. Samples were then taken periodically. The methane and ethane concentrations in the headspace gas samples were measured using a GC-FID (Agilent HP 6890 Gas Chromatograph, Hewlett-Packard), equipped with an HP-PLOT Al_2_O_3_/KCl column (polar alumina phase deactivated with potassium chloride, length, 50 m; diameter, 0.32 mm, thickness, 8 µm). The headspace gas samples (100 µl) were injected with a gastight 1700 SampleLock Syringe (PN81056, Hamilton). A calibration curve was generated using methane standards. The ratio of the peak areas generated by methane and ethane for the same concentration each was used to quantify ethane. The limit of detection (LOD = 0.03 nmol) and limit of quantification (LOQ = 0.1 nmol) for ethane were determined using signal to noise ratio of 3.3 and 10, respectively.

## Results and discussion

### Ethane production in vivo and via resting cell suspensions

**Fig. 2:**
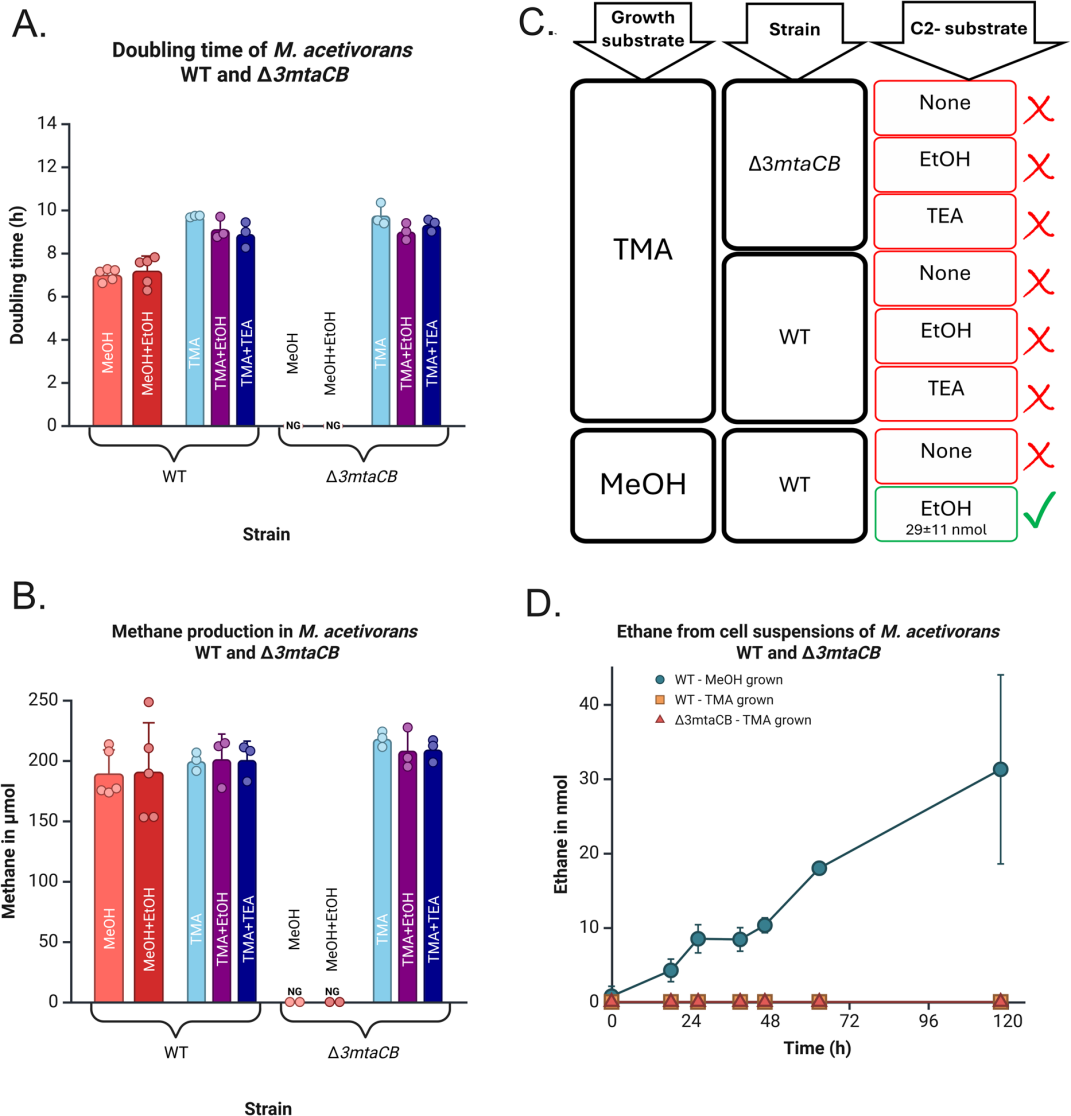
Influence of ethylated substrates on *M. acetivorans* WT and ∆*3mtaCB*. **A** Doubling time. The presence of ethylated substrates (EtOH and TEA) did not significantly affect the doubling time of the WT and *∆3mtaCB* strains significantly (one-way ANOVA; p > 0.05). **B** Methane yield after 216 h incubation. Ethylated substrates (EtOH and TEA) did not affect the methane yield (one-way ANOVA; p > 0.05). **C** Requirements for ethane production. Red boxes and crosses signify that ethane was below level of quantification (LOQ = 0.1 nmol), whereas the green box and the check mark signifies formation of ethane. ± signifies standard deviation. **D** Cell suspension experiments with EtOH. Only cell suspensions of the MeOH-grown *M. acetivorans* WT strain showed ethane production at a rate of 0.26 nmol/h. Ethane was below LOQ for the cell suspensions of the TMA-grown WT and *∆3mtaCB* strains. Data was obtained from three independent cultures but five independent cultures for the doubling time and methane yield of the WT strain with MeOH and MeOH + EtOH. NG: No growth. Error bars indicate the standard deviation. Raw data and statistical analyses are available as SI. Created in https://BioRender.com

Growth of *M. acetivorans* WWM1 (WT) and WWM13 (*∆3mtaCB*) was compared in presence of ethanol over 216 h. WT was grown with methanol (MeOH), methanol and ethanol (MeOH + EtOH), trimethylamine (TMA), trimethylamine and ethanol (TMA + EtOH) and trimethylamine and triethylamine (TMA + TEA), whereas *∆3mtaCB* could only grow with TMA, TMA + EtOH and TMA + TEA. The strain and media composition information are available in Table 1 and Table 2. The presence of ethylated substrates did not affect the doubling time of the WT or the *∆3mtaCB* strains (Fig. 2A). The lack of change in methane yield (Fig. 2B) shows that ethylated substrates were not oxidized at the same time as MeOH or TMA, which would have led to additional reducing power available leading to increased methane production. The reducing equivalents required for ethane production could be sourced from an intracellular electron donor such as glycogen that has been shown to be accumulated in *M. acetivorans* (Santiago-Martínez et al. 2016). Ethane was produced only in ethanol-supplemented medium and when methanol-specific methyltransferases are present and expressed (Fig. 2C). Ethane production was below LOQ from TEA suggesting only methanol-specific methyltransferases are capable of promiscuous ethyltransfer (Fig. 2C). The lack of ethane in the absence of ethanol or absence of *mtaCB*s shows their necessity for ethane formation.

Cell suspensions of *M. acetivorans* WT (grown with methanol) produced ethane when supplemented with ethanol at a rate of 0.26 nmol/h in 118 h of incubation (as calculated using linear regression), whereas ethane was below LOQ from cell suspensions of WT and *∆3mtaCB* grown with TMA (Fig. 2D). Growth with TMA suppresses the expression of methanol-specific methyltransferases, but MtaCB3 is transiently expressed while growing on TMA but only after TMA and DMA is consumed to form MMA (Bose et al. 2006). As the resting cell suspensions contained a protein synthesis inhibitor, this transient expression of MtaCB3 would be blocked and hence no detectable ethane production was obtained even in *M. acetivorans* WT cells grown with TMA. The ethane produced by WT cell suspensions (grown with methanol) in presence of protein synthesis inhibitor shows that the proteome present in *M. acetivorans* WT during growth on methanol is indeed responsible to give the microbe the capacity for ethane production. *M. acetivorans ∆3mtaCB* also failed to show ethane production in presence of ethanol as the strain lacks all three *mtaCB*s.

### Testing the substrate promiscuity of Mta isozymes in vivo and via resting cell suspensions

**Fig. 3:**
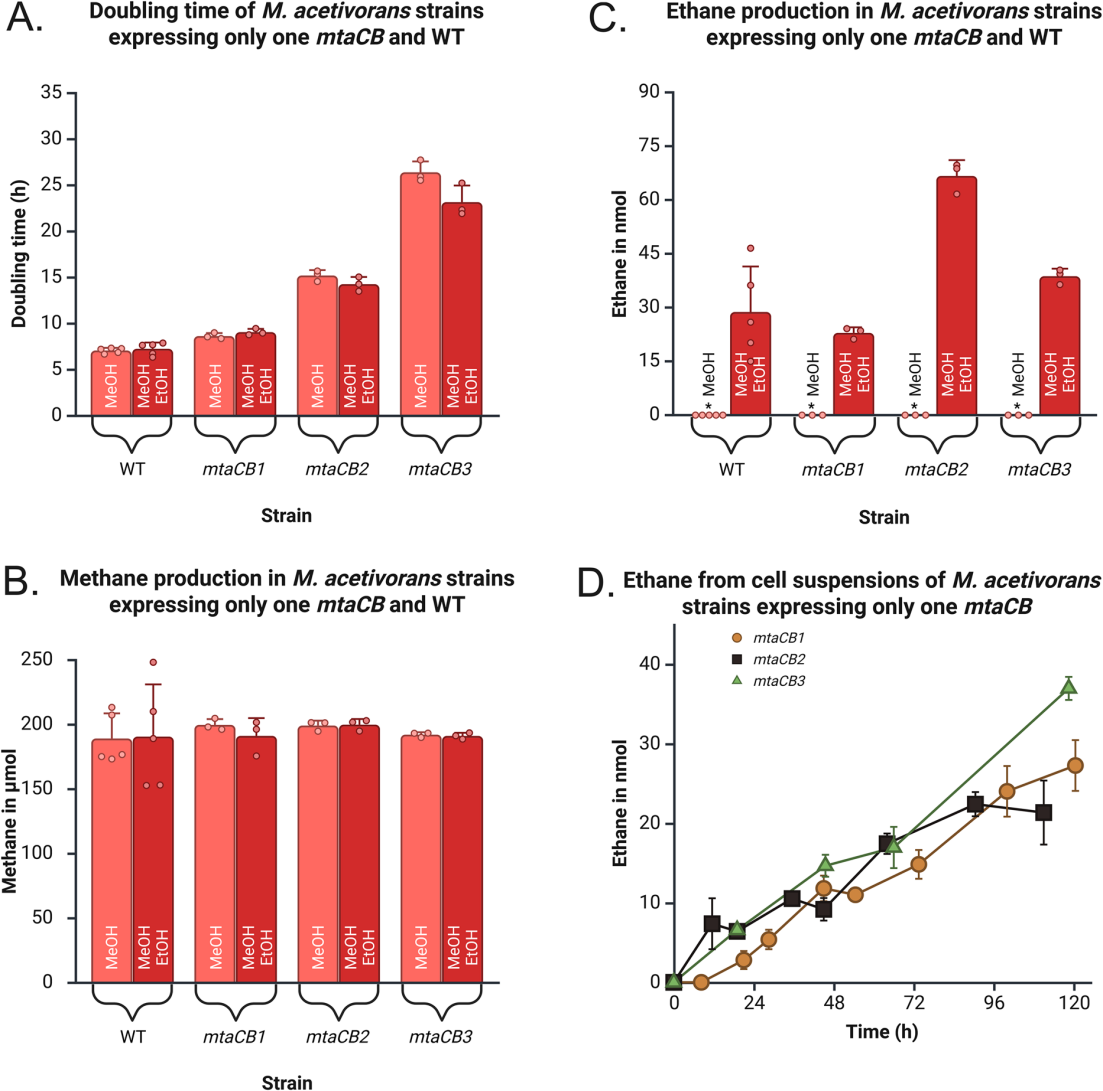
Influence of ethanol on *M. acetivorans* double deletion strains that express only *mtaCB1, mtaCB2 or mtaCB3*. **A** Doubling time. **B** Methane yield after 285 h incubation. Ethanol did not affect the doubling time or the methane yield (one-way ANOVA; p > 0.05). **C** Ethane yield after 285 h incubation. Ethane production was seen from each strain in presence of ethanol (one-way ANOVA; p < 0.001). Data of WT strain shown for comparison from Fig. 2. *: Below LOQ = 0.1 nmol. **D** Cell suspension experiments with EtOH. Cell suspensions of all three strains- *mtaCB1*, *mtaCB2* and *mtaCB3* showed ethane production at a rate of 0.24 nmol/h, 0.19 nmol/h and 0.3 nmol/h respectively. Data was obtained from three independent cultures of the double deletion strains whereas five independent cultures of the WT. The incubation period for WT strain was 216 h. Error bars indicate the standard deviation. Raw data and statistical analyses are available as SI. Created in https://BioRender.com

Because *M. acetivorans* encodes three different isozymes of MtaCB, we tested the activation of ethanol by each isozyme. *M. acetivorans* double deletion strains capable of expressing only one isozyme were obtained from a previous study (Pritchett and Metcalf 2005). All three double deletion strains were grown over 285 h (longer than WT or *∆3mtaCB* as they grow slower) and with MeOH as growth substrate and EtOH provided where necessary (Fig. 3A, B). The presence of ethanol did not affect the doubling time or the methane yield of all three double deletion strains (one-way ANOVA; p > 0.05).

Each strain was capable of ethane production in presence of ethanol (Fig. 3C), showing that all three isozymes can activate ethanol for subsequent ethyl transfer. At the end of 285 h of incubation, *mtaCB2* had produced the highest amount of ethane (one-way ANOVA, p < 0.01). Like the WT and *∆3mtaCB* strains, cell suspensions of the *mtaCB1*, *mtaCB2* and *mtaCB3* strains were tested for ethane production from ethanol in the presence of a protein synthesis inhibitor. All three cell suspensions of *mtaCB1*, *mtaCB2* and *mtaCB3* could produce ethane at a rate of 0.24 nmol/h, 0.19 nmol/h, and 0.3 nmol/h, respectively (Fig. 3D, linear regression). The aim of the cell suspension experiment was to show capability of each isozyme to enable the microbe to produce ethane. The strain *mtaCB3* had a higher rate than the *mtaCB1* or *mtaCB2* strains, but that did not lead to higher ethane accumulation in growing cells (Fig. 3C). The difference in accumulation of ethane in growing cells compared to the rate of ethane production occurs because production of ethane begins after all methanol is consumed in the media, which is slowest for *mtaCB3* (Fig. S1) (Belay and Daniels 1988; Pritchett and Metcalf 2005). Specific ethane production rates for each isozyme cannot be given due to the complex regulation of *mtaCB* operons (Bose et al. 2006).

To quantify ethanol activation to ethylated MtaC directly by purified MtaB, we heterologously expressed MtaB1-3 individually in *E. coli*. We could not obtain active enzyme even though the proteins were well folded and showed high thermal stability up to 60 °C (see supplementary materials for further discussion and characterization of the purified enzymes).

### In vitro ethane formation from ethanol, ethylcobalamin and ethyl-CoM using *M. acetivorans* WT cell lysate

**Fig. 4:**
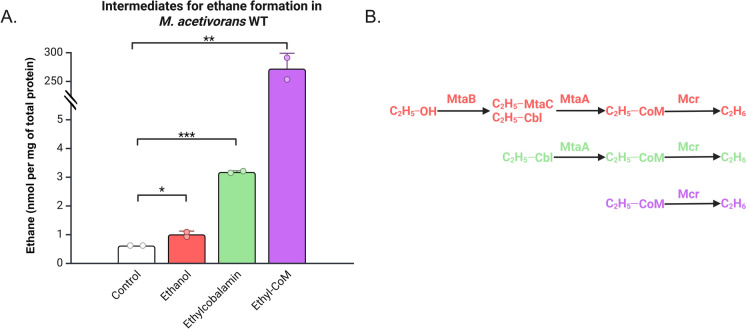
Intermediates for ethane formation in *M. acetivorans* WT cell lysate measured after 24 h. **A** Ethanol, ethylcobalamin and ethyl-CoM were tested as substrates for ethane production in *M. acetivorans* WT cell lysate. All three substrates showed ethane production that was significantly higher than control which was without any ethylated substrate (unpaired T-test; *, p < 0.05; **, p < 0.01; ***, p < 0.001). The amount of ethane produced from ethyl-CoM was scaled in the final bar. The amount of ethane produced was normalized to the total protein added. Data was obtained from duplicates. Error bars represent standard deviation. Raw data and statistical analyses are available as SI. **B** Confirmed intermediates for ethane production. Each reaction corresponds to the bar of same color in Fig. 4A, highlighting the enzymes involved. Cobalamin (Cbl) was used as an alternative for the corrinoid protein MtaC. Increasing ethane production from ethanol, ethylcobalamin and ethyl-CoM is in line with the hypothesis proposed in Fig. 1. Created in https://BioRender.com

To corroborate the role of Mta for ethane production, we utilized *M. acetivorans* WT cell-lysate to probe for ethane formation from different intermediates of the pathway parts (Figs. 1, 4A and B). The reaction cascades were set up in a reverse sequential way by first confirming that ethyl-CoM is converted to ethane. Next, we tested ethyl-cobalamin as a surrogate for ethyl-MtaC and demonstrated the cascade to ethane. Finally, we tested ethanol. Ethane was measured after 24 h incubation. Significant ethane production could be seen from ethanol, ethylcobalamin and ethyl-CoM when compared to control which did not contain any added ethylated substrate. The amount of ethane produced matches the pathway in reverse with highest ethane produced from ethyl-CoM, then ethylcobalamin, and finally ethanol. These results are in line with the hypothesis in Figs. 1 and 4B, because ethane formation from ethanol relies on the substrate promiscuity of three enzymes (Mcr, MtaA, and MtaB) whereas ethane formation from ethylcobalamin and from ethyl-CoM relies on the substrate promiscuity of only two (Mcr and MtaA) or only one enzyme (Mcr), respectively. However, the lower amounts of ethane produced from ethanol and ethylcobalamin could also stem from the use of cobalamin. Either because cobalamin is not the native corrinoid in *Methanosarcina* (Pol et al. 1982), or due to the lack of protein-bound corrinoids (MtaC), even though MtaB has been shown to accept non-protein-bound cobalamin as substrate (Sauer et al. 1997).

## Conclusion

The ethanol-to-ethane metabolism in *M. acetivorans* requires ethyl-group transfer via substrate promiscuity by Mta, because *M. acetivorans* ∆*3mtaCB*, that has all *mtaCB* genes deleted, was unable to produce ethane under any condition tested. The three requirements for in vivo ethane production are: (1) presence of *mtaCB* genes, (2) growth on methanol to ensure expression of *mtaCB* genes and (3) presence of ethanol in the culture medium. All three isozymes of the methanol-specific methyltransferase showed capability of substrate promiscuity; however, the ethane yields were ca. 3 orders of magnitude lower compared to methane yields. Our work demonstrates that ethyl transfer reactions via corrinoid-dependent enzymes are possible and should be considered as additional reactions in metabolic networks, reaffirming previous reports suggesting ethanol catalyzed by methanogens as a possible source of biogenic ethane (Oremland 1981; Belay and Daniels 1988). Corrinoid-dependent transfer of higher alkyl-groups may be important for anaerobic alkane oxidizers where the pathway for alkane oxidation beyond alkyl-CoM is unknown, but has been hypothesized to involve corrinoid-dependent alkyl transfer (Wegener et al. 2022). While the rates of ethyl-transfer rely only on substrate promiscuity in *M. acetivorans*, this reaction may provide a template for evolution and could have led to corrinoids that efficiently transfer ethyl-groups.

## Supplementary Information

Below is the link to the electronic supplementary material.

Supplementary file1 (XLSX 232 KB)

Supplementary file2 (DOCX 1881 KB)

## Data Availability

All other study data are available in the published article and/or the Supporting information.
