# Supplementary material for: Methanosarcina acetivorans requires methanol:coenzyme M methyltransferases for ethane formation from ethanol
Source: Antonie Van Leeuwenhoek. 2025 Sep 20;118(10):154. doi: 10.1007/s10482-025-02165-x (PMC12450230; doi:10.1007/s10482-025-02165-x)
Supplement: Supplementary file 2 — Supplementary file2 (DOCX 1881 KB) [file 10482_2025_2165_MOESM2_ESM.docx]

**Supplementary information**

**Methods**

**Protein expression and purification.** Plasmids, encoding N-terminal His_6_-tagged MtaB1-3, were transformed into chemical competent *E. coli* BL21 (DE3) and plated on LB agar, supplemented with 100 µg/mL. Liquid cultures were prepared by inoculating a single bacterial colony in 5 mL LB media supplemented with 100 µg/mL ampicillin. Inoculated 1 L LB_AMP_ was incubated at 37 °C and induced with 100 µM IPTG at OD_600_ = 0.6 - 0.8. Protein expression took place at 25 °C for 16 hours. Cells were harvested by centrifugation (5000 x g, 15 min, 4 °C) and freeze in liquid nitrogen. For purification, cell pellets were resuspended on ice using lysis buffer (50 mM sodium phosphate (pH 7.4), 300 mM NaCl, 10% glycerol, supplemented with protease inhibitor mix (Sigma Scientific), DNase I, and lysozyme). Cells were lysed by sonication (30% power, 1 s pulse, 1 s pause, 3 cycles) and cell debris were removed by centrifugation (20000 x g, 1 h, 4 °C). The resulting supernatant was transferred to 1 mL Nickel-NTA agarose equilibrated with lysis buffer and mixed for 1 h at 4 °C with gentle rocking. The protein-bound resin suspension was transferred to a gravity flow column (Bio-Rad Econo-Pac-chromatography column) and the resin was washed with 40 column volumes (CV) 50 mM sodium phosphate buffer (pH 7.4), 20 mM imidazole, 300 mM NaCl, 10% (v/v) glycerol, and with 20 CV 50 mM sodium phosphate buffer (pH 7.4), 40 mM imidazole, 300 mM NaCl, 10% (v/v) glycerol. 4 mL of lysis buffer and 200 µL of (240 µM) HRV-3C protease were added to the resin, mixed and incubated for 16 h at 4 °C to cleave His_6_-tag. The flow-through, which contained tag-free protein, was collected and the resin was washed with 5 x 1 CV lysis buffer. Protein concentration was determined by UV-Vis at 280 nm. Sodium dodecyl sulfate polyacrylamide gel electrophoresis (SDS-PAGE) was used to confirm protein purity.

**Circular dichroism spectroscopy.** Circular dichroism (CD) spectroscopy was performed to assess the secondary structure and thermal stability of the protein samples. Measurements were carried out using a JASCO J-1500 CD spectrometer, equipped with a Peltier temperature control system, and a 0.1 cm pathlength quartz cuvette. Protein samples were prepared at a final concentration of 5 µM in 5 mM sodium phosphate buffer, pH 7.4, containing 15 mM NaCl and 1% glycerol. CD spectra were recorded in the far-UV range (190–260 nm) with a scan speed of 50 nm/min, a bandwidth of 1 nm, and a data pitch of 1 nm. Each spectrum was the average of three accumulations and was corrected for baseline contributions by subtracting the corresponding buffer spectrum. To evaluate thermal stability, CD spectra were recorded at 2 °C intervals from 20 °C to 60 °C, with an equilibration time of 30 seconds at each temperature. After reaching the maximum temperature, samples were cooled back to 20 °C, and spectra were recorded again to assess the reversibility of protein folding. All measurements were performed in technical triplicate, and spectra were analyzed using Spectra Manager software (JASCO) and Microsoft excel.

**Results and Discussion**

**
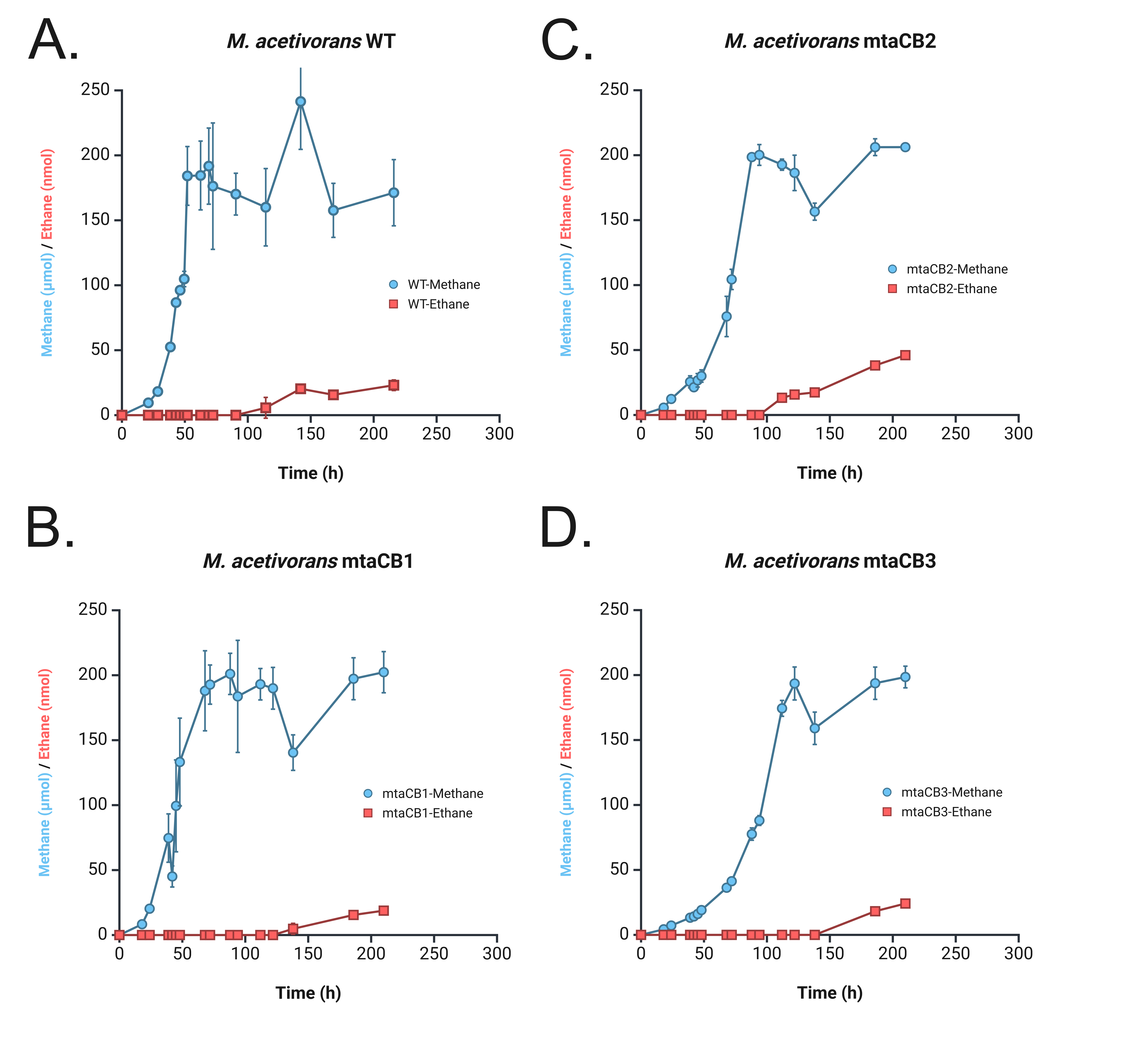
Figure S1: Methane and ethane production in WT and double deletion strains growing on MeOH+EtOH. (A)** WT strain **(B)** mtaCB1 strain **(C)** mtaCB2 strain **(D)** mtaCB3 strain. For all strains tested, ethane production begins after methane production has ceased reaching the maximum theoretical yield of ca. 225 µmoles. Data was obtained from duplicates for the WT strain and triplicates for the double deletion strains. Error bars represent the standard deviation. Raw data is available as an additional SI file.

**
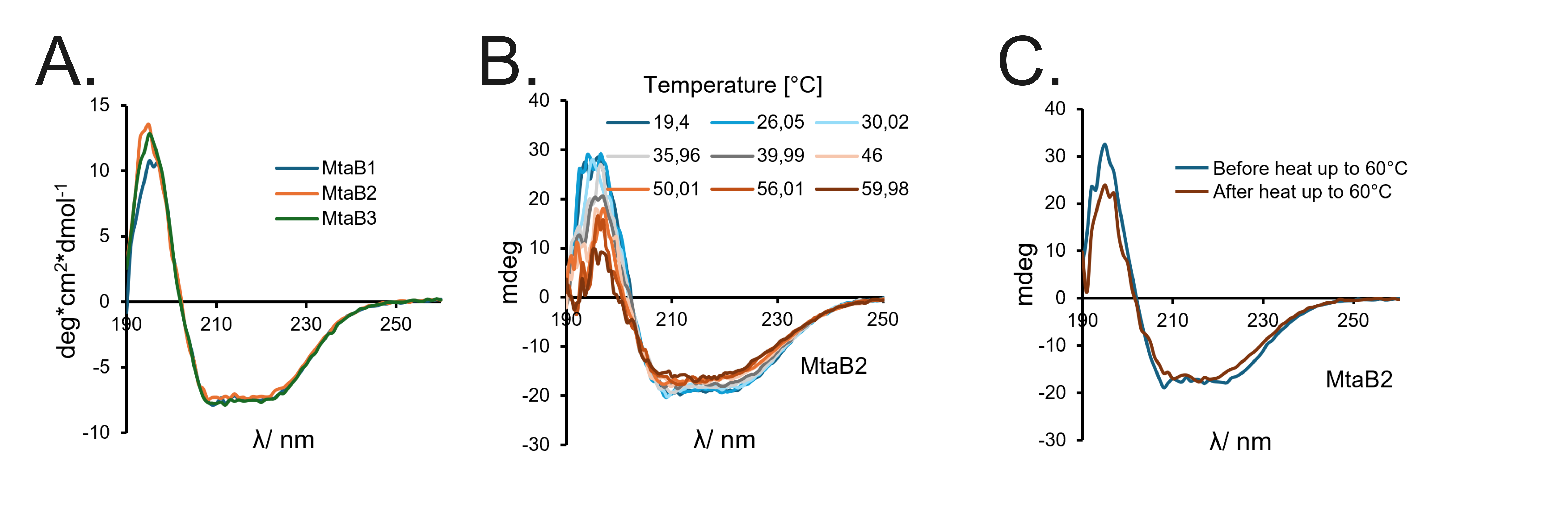
Figure S2: Circular dichroism spectroscopy (CD) of heterologously expressed and purified MtaB1-3 indicating heat stable protein structures. (A)** CD spectra of purified MtaB1-3 isoforms show high structural similarity, suggesting conserved secondary structure elements. **(B)** Thermal stability of MtaB2 was assessed by recording CD spectra at 2 °C intervals up to 60 °C. The spectra show minimal variation of secondary structure elements, indicating preserved secondary structure upon heating. **(C)** After heating to 60 °C, the MtaB2 sample was cooled back to 20 °C. CD spectra recorded at 20 °C before and after heating are nearly identical, suggesting reversible folding and thermal stability. Measurements were performed in technical triplicate with three accumulations each, using JASCO CD J-1500 instrument. Protein concentration was 5 µM in 5 mM sodium phosphate buffer (pH 7.4), 15 mM NaCl, and 1% glycerol.

**Heterologous expression and purification of MtaB1-3.** The MtaB1-3 proteins were successfully purified with an N-terminal His_6_-tag, following established protocols (Sauer and Thauer 1997). Structural analysis using CD spectroscopy confirmed that the proteins were well-folded (Fig. S2A) and maintained stability when heated to 60 °C (Fig. S2B). Refolding experiments demonstrated that the proteins could return to their original fold after being heated to 60 °C (Fig. S2C). Despite the proteins being well-folded and thermally stable, no MtaB activity was detected. This lack of activity might be due to deactivation of heterologously expressed proteins under the assay conditions tested or a short activity lifespan, which does not necessarily correlate with protein stability. Similar findings have been reported for MtaB from *M. barkeri*, which was only active for 5 minutes before deactivation (Sauer et al. 1997). Additionally, a high variability in specific activity between different preparations of *M. barkeri* MtaB was noted (Sauer and Thauer 1999), highlighting the challenges associated with handling this enzyme.

**References**

Sauer K, Harms U, Thauer RK (1997) Methanol: Coenzyme M Methyltransferase from *Methanosarcina Barkeri*: Purification, Properties and Encoding Genes of the Corrinoid Protein MT1. European Journal of Biochemistry 243:670–677. https://doi.org/10.1111/j.1432-1033.1997.t01-1-00670.x

Sauer K, Thauer RK (1997) Methanol: Coenzyme M Methyltransferase from *Methanosarcina Barkeri*: Zinc Dependence and Thermodynamics of the Methanol:Cob(I)alamin Methyltransferase Reaction. European Journal of Biochemistry 249:280–285. https://doi.org/10.1111/j.1432-1033.1997.t01-1-00280.x

Sauer K, Thauer RK (1999) Methanol:coenzyme M methyltransferase from *Methanosarcina barkeri* ‐‐ substitution of the corrinoid harbouring subunit MtaC by free cob(I)alamin. European Journal of Biochemistry 261:674–681. https://doi.org/10.1046/j.1432-1327.1999.00355.x
